# Impact of SARS-CoV-2 infection on the recovery of peripheral blood mononuclear cells by density gradient

**DOI:** 10.1038/s41598-021-83950-2

**Published:** 2021-03-01

**Authors:** Maria D. I. Manunta, Giuseppe Lamorte, Francesca Ferrari, Elena Trombetta, Mario Tirone, Cristiana Bianco, Alessandra Cattaneo, Luigi Santoro, Guido Baselli, Manuela Brasca, Mahnoosh Ostadreza, Elisa Erba, Andrea Gori, Alessandra Bandera, Laura Porretti, Luca V. C. Valenti, Daniele Prati

**Affiliations:** 1 Department of Transfusion Medicine and Hematology, Milano Cord Blood Bank, Processing Facility and Biobank POLI-MI, Fondazione IRCCS Ca’ Granda Ospedale Maggiore Policlinico, Via Francesco Sforza, 35, 20122 Milan, Italy; 2 Flow Cytometry and Cell Sorting Laboratory, Analysis Laboratory, Fondazione IRCCS Ca’ Granda Ospedale Maggiore Policlinico, Milan, Italy; 3 Directorate of Allied Health Professions, Fondazione IRCCS Ca’ Granda Ospedale Maggiore Policlinico, Milan, Italy; 4 Infectious Diseases Unit, Fondazione IRCCS Ca’ Granda Ospedale Maggiore Policlinico, Milan, Italy; 5 Centre for Multidisciplinary Research in Health Science (MACH), Università degli Studi di Milano, Milan, Italy; 6 Department of Pathophysiology and Transplantation, Università degli Studi di Milano, Milan, Italy

**Keywords:** Medical research, Biological techniques

## Abstract

SARS-CoV-2 virus infection is responsible for coronavirus disease (COVID-19), which is characterised by a hyperinflammatory response that plays a major role in determining the respiratory and immune-mediated complications of this condition. While isolating peripheral blood mononuclear cells (PBMCs) from whole blood of COVID-19 patients by density gradient centrifugation, we noticed some changes in the floating properties and in the sedimentation of the cells on density medium. Investigating this further, we found that in early phase COVID-19 patients, characterised by reduced circulating lymphocytes and monocytes, the PBMC fraction contained surprisingly high levels of neutrophils. Furthermore, the neutrophil population exhibited alterations in the cell size and in the internal complexity, consistent with the presence of low density neutrophils (LDNs) and immature forms, which may explain the shift seen in the floating abilities and that may be predictive of the severity of the disease. The percentage of this subset of neutrophils found in the PBMC band was rather spread (35.4 ± 27.2%, with a median 28.8% and IQR 11.6–56.1, Welch’s *t*-test early phase COVID-19 versus blood donor healthy controls *P* < 0.0001). Results confirm the presence of an increased number of LDNs in patients with early stage COVID-19, which correlates with disease severity and may be recovered by centrifugation on a density gradient together with PBMCs.

## Introduction

The novel severe acute respiratory syndrome coronavirus 2 (SARS-CoV-2) is an enveloped single-stranded RNA virus belonging to the subfamily Coronavirinae in the family of Coronaviridae of the order Nidovirales^1^. It is the etiologic agent of the severe acute respiratory syndrome^2^ named Corona Virus Disease (COVID)-2019. SARS-CoV-2 virus binds the Angiotensin-converting enzyme 2 (ACE2) as receptor for cell entry and the transmembrane protein serine protease TMPRSS2 for S protein priming^3,4^. One peculiarity of SARS-CoV-2 is represented by its particular tropism for type I and type II pneumocytes, alveolar macrophages and mucus cells in the nasal cavity^5^, although beyond the respiratory disease, systemic disorders have also been reported^6^. ACE2 enzyme is widely expressed on the plasma membranes of various cell types in different tissues, including intestinal and endothelial cells^7–9^. The vascular endothelium is among the tissues affected by the SARS-CoV-2 infection as the normal ACE2 peptidase activity leads, through the G-protein coupled receptor, to the activation of signalling pathways ultimately responsible for vasodilation, anti-inflammatory and anti-fibrotic responses. Endothelial cell dysfunction, endotheliitis, vascular damage, vasculitis as well as Kawasaki-like syndrome and Takotsubo cardiomyopathy have been reported by us and others^10–15^.

The cytokines storm^16–18^ and the impaired interferon response have been found in severe COVID-19 patients^19–21^. The viral inhibition of type I and type III interferons, previously described for Middle East Respiratory Syndrome (MERS)^22^ may be important. However conclusive studies on the immune response to SARS-CoV-2 infection are still underway. Coagulation factors, antibodies or complement on the surface of red cells and antiphospholipid antibodies may also play a role in the complicated puzzle of the COVID-19 immune response^10,23–28^. The development of autoimmune responses can contribute to the severity of COVID-19^29,30^ and on the other hand, the occurrence of autoantibodies in viral infections is well-established^31–36^. The contribution of cell-mediated immunity, both innate and acquired, is yet unclear, though lymphopenia appears to be one of the main features of this viral disease^37,38^ and also the presence of an atypical monocyte population^39^. It has been also reported that increased levels of neutrophil‐to‐lymphocyte ratio (NLR) reflecting an enhanced systemic inflammation may predict the clinical severity and suggest a poor prognosis^40,41^.

Peripheral Blood Mononuclear Cells (PBMCs) are an attractive tissue source for immunological, molecular and pharmacogenomic studies. PBMCs from peripheral blood consist in a heterogeneous cell population with a round nucleus (i.e. lymphocytes, monocytes, natural killer cells (NK cells) or dendritic cells). It has been shown that PBMCs are permissive to SARS-CoV infection and support viral replication^42–44^. These cell populations have been used for transcriptomics evaluations in the current pandemic^45–47^.

Sedimentations on density gradient are well-established techniques to isolate cells and cellular organelles^48–55^. The sedimentation rate of the cells strongly depends on their size^55^. Lymphoprep™ density gradient medium, which has a density of 1.077 g/ml, is widely used for the isolation of mononuclear cells from human blood. After centrifugation, due to their lower buoyant density, mononuclear cells (i.e. lymphocytes and monocytes) can be recovered, at the interface between the sample and the density medium, while granulocytes (or polymorphonuclear cells, PMNs) and erythrocytes are usually found at the bottom of the centrifuge tube. According to Graham^49^ monocytes, being larger (15–20 µm), sediment to a slightly lower density (ρ = 1.064 g/ml) while lymphocytes, which are smaller (6–20 µm), deposit at a density of ρ = 1.072 g/ml. Thus, the peripheral blood monocytes usually form a sharp cloudy band above the lymphocyte ring. Instead, the granulocytes, which have a relative higher density, penetrate through the isosmotic medium and sediment above the red blood cells layer. PMNs in the bloodstream refer to neutrophils, eosinophils and basophils. PMNs and especially neutrophils are resolved in a gradient of density around 1.088 g/ml^49,55^. Neutrophils are the most abundant circulating phagocytes and their activity is exerted through the release of soluble cytotoxic proteins and peptides from their granules, through the production of reactive oxygen species by a membrane-bound NADPH oxidase and by the formation of neutrophil extracellular traps (NETs)^56–58^. Neutrophils are short-lived, front-line effectors of acute inflammation and first responders against a wide range of pathogens including bacteria, fungi, and protozoa. As they are quickly recruited at the inflammatory site, neutrophils have been latterly implicated in the antiviral immune response^59^. It has been recently reported that neutrophils with immunomodulatory capabilities (i.e. augmented chemotaxis, increased bacterial confinement and ability to suppress T-cell proliferation) can shift to a low buoyant density^60^. However prolonged activation of neutrophils can have detrimental effects contributing to lung injuries^61^, the NET formation may play a crucial role in the exacerbation of the lung disease and in other complications associated with COVID-19 as well as in the mortality of COVID-19 patients^62^.

However, neutrophils display a morphological, phenotypical, functional heterogeneity and a subset of these cells, defined as low density neutrophils (LDNs), have the ability to partition with PBMC fraction after density gradient centrifugation^59^. LDNs showed in vitro increased activation markers comparable to those seen in patients with chronic inflammatory diseases^59^. The appearance of immature neutrophils or LDNs in COVID-19 patients has also been postulated by Wilk et al. and by Schulte-Schrepping et al.^46,47^.

## Results

### Density gradient qualitative evaluation

**Figure 1:**
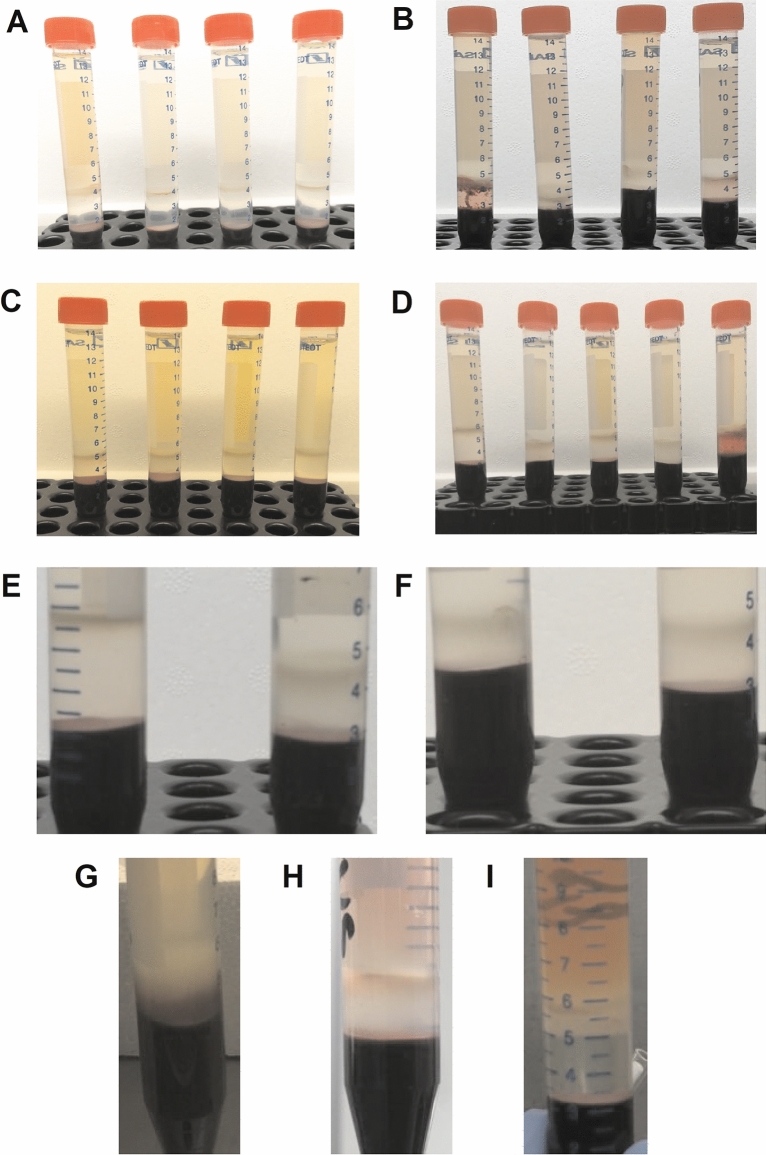
Density gradient images of samples from different cohorts. Healthy controls are shown in panel (**A**), while in panel (**B**) samples collected within 24 h after hospitalisation are represented. Panel (**C**) displays samples of recovered COVID-19 patients (14 d after discharge). The specimens in panel (**D**) are processed at the same time and under the same conditions: the first sample (left) belongs to a recovered COVID-19 patient (14 d post), whereas the other four refer to the 1st COVID-19 group (within 24 h). Panel E displays magnified images from a sample of the recovered group (left) and one of the early phase COVID-19 cohort (right). Enlarged images of two different samples, both collected at 24 h from hospitalisation, are shown in panel (**F**). Panels (**G**–**I**) represent samples from early phase COVID-19 patients with double bands on top of the erythrocytes cushion.

Patients were stratified according to their clinical status and the specimens were grouped in accordance with the time of sampling i.e. within 24 h from admission (early phase COVID-19) or 14 days after discharge. Healthy control samples were provided by blood donors at the time of the donation. As expected, the typical mononuclear population from control blood donors was recovered at the interface between the density medium and the sample (Fig. 1A). By contrast, the vast majority of PBMCs derived from patients with an early phase of COVID-19 floated quite differently. Some of these samples showed a thicker and fluffier band of mononuclear cells, mostly lower than usual, sometimes stickier, often decorated underneath by a dispersed fine crown of red blood cells or with erythrocyte aggregates (Fig. 1B). A number of times the density medium also appeared rather cloudy or less transparent than usual. The buoyant density was restored in PBMCs obtained from blood samples collected from patients at 14 d post-discharge (recovered COVID-19 cohort; Fig. 1C), although a slightly thicker band was often observed. Comparison between one sample at day 14 post-discharge and four samples collected at the admission, processed simultaneously and under the same conditions, are shown in Fig. 1D. For a better appreciation, magnification of a 14 d post-discharge sample (recovered COVID-19 cohort) and a sample taken within 24 h from admission (early phase COVID-19 cohort) are displayed in Fig. 1E, while Fig. 1F is a closer view of two samples taken at the time of admission into hospital. Occasionally two bands, one right at the interface between the sample and the medium, and a second within the density medium itself, were also observed (Fig. 1, panels G, H and I).

### Population dynamics study

An automated haematology cell analyser was used to assess the cell populations (neutrophils, lymphocytes, monocytes) present in the band isolated on the density gradient. To have an estimate of the variation in the cell composition in the collected fraction we determined also the cell ratios.

**Figure 2:**
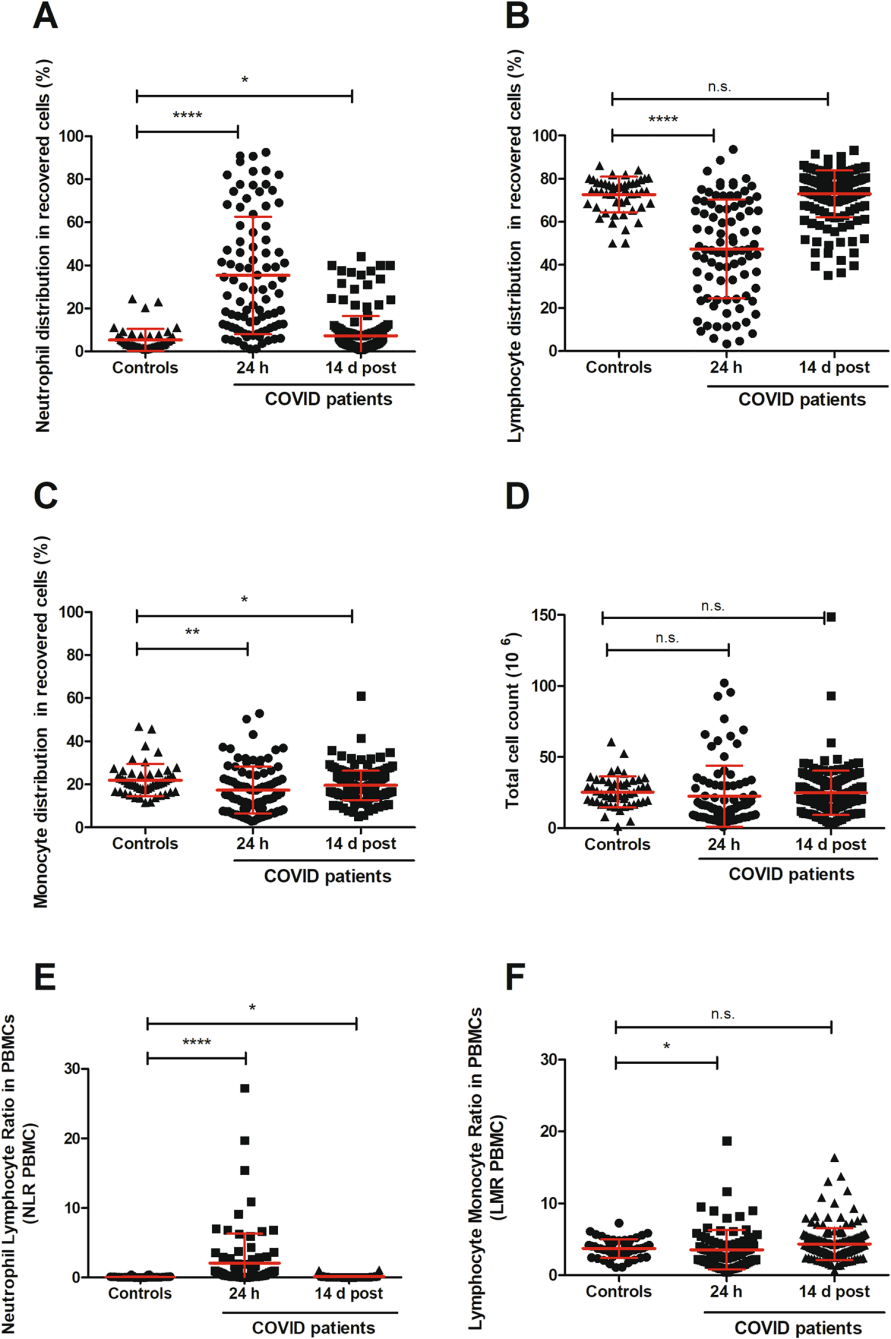
Enriched cell populations recovered on density gradient. Panel (**A**) represents neutrophil populations from the healthy blood donors (N = 50) and from the two COVID-19 cohorts: early phase (24 h; N = 90) and recovered (14 d post-discharge; N = 174). Panel (**B**)shows the lymphocytes while in panel (**C**) the monocytes are displayed. The total cell counts of all three groups, providing an estimate of the cell enrichments, are indicated in panel (**D**). The neutrophil-to-lymphocyte ratio (NLR-PBMC) of cells recovered after gradient separation is shown in panel (**E**), while lymphocyte-to-monocyte ratio (LMR-PBMC) of the same cell density fraction is displayed in panel (**F**). Each dot represents a patient sample and the values are expressed as mean ± standard deviation (SD). Statistically significant differences were expressed as *for *P* ≤ 0.05, **for *P* ≤ 0.01, ***for *P* ≤ 0.001 and ****for *P* ≤ 0.0001, while n.s. signifies not statistically significant for *P* > 0.05.

The neutrophils in the samples of early phase of COVID-19 (taken within 24 h from hospitalisation) heavily outnumbered in percentage those of the convalescent patients (14 d after discharge) as well as those from healthy controls (Fig. 2A). In patients from the 24 h cohort, the amount of neutrophils found in the PBMC band was of 35.4 ± 27.2% (mean ± SD; *t*-test versus healthy controls *P* < 0.0001), with a median value of 28.8% (IQR 11.6–56.1), thus indicating that the number of neutrophils varied extensively among the different COVID-19 patients, probably depending from the severity of the disease at the time of hospitalisation. The neutrophil distribution data was less spread out in the cohort of patients who had the samples taken 14 days after being released from hospital. The mean value in the discharged patients group was 7.4 ± 9.2% (*t*-test versus blood donor controls *P* = 0.0469; median of 3.8%; IQR 2.5–7.9) while a range much narrower was displayed by the healthy controls i.e. 5.4 ± 5.2% (with a median of 3.3%; IQR 2.3–6.8).

Conversely, the percentage of lymphocytes showed an opposite trend across the different cohorts as compared to what mentioned above for the neutrophil populations (Fig. 2B). Indeed, at admission, the average percentage of the lymphocytes displayed a mean value of 47.3 ± 2.4% (*t*-test vs healthy controls *P* < 0.0001; median 46.9%; IQR 28.2–67.4), while the samples from post-discharged patients showed an average value of 73.0 ± 10.8% (*t*-test vs blood donor controls *P* = 0.8120; median 74.7%; IQR 70.1–80.1) and the mean value for the lymphocytes from healthy blood controls was 72.6 ± 8.3% (median 74.3%; IQR 68.0–78.2). Thus, the percentage of lymphocytes, collected post-gradient, returned to normal levels at 14 d post-discharge as suggested by the comparison with the values found for the blood donors.

Although still statistically meaningful, the differences in the monocyte population among the three groups were less remarkable (Fig. 2C). The pattern of monocyte distribution, expressed as mean percentage was 17.3 ± 10.9% (Welch’s *t*-test vs healthy controls *P* = 0.0034; median 15.0%; IQR 8.4–23.0) for the early phase COVID-19 cohort (i.e. samples within 24 h from hospital admission) and 19.6 ± 6.9% (*t*-test vs blood donor controls *P* = 0.0447; median 18.9%; IQR 15.2–23.2) for the COVID-19 recovered group, whereas healthy controls showed a percentage of 22.0 ± 7.5% (median 20.5%; IQR 16.7–24.6).

To determine the efficiency of the cell enrichment in the PBMC fraction from the samples of the three different cohorts, we measured the total cell counts that somehow provide the yield of the separation method. The total number of cells recovered from early phase COVID-19 patients was 22.6*10^6^ ± 21.5 (*t*-test vs blood donor controls *P* = 0.2935; median 15.4; IQR 8.8–29.9), while 24.9*10^6^ ± 15.5 cells (*t*-test vs healthy controls *P* = 0.7595: median 22.4; IQR 15.8–32.8) were collected in samples from 14 d post-discharge patients and 25.5*10^6^ ± 10.9 cells (median 24.2; IQR 18.4–32.2) were retrieved from the healthy control samples (Fig. 2D).

The enrichment in terms of total number of cells recovered was not too dissimilar in the three groups and an optimal yield of the mononuclear cells should not account for more than 5% of granulocytes. To assess the efficiency of the recovery of the PBMCs after the isolation and to evaluate the distribution pattern of the prevailing cell types post-separation through the density gradient, we determined the neutrophil-to-lymphocyte ratio (NLR-PBMC, Fig. 2E) and lymphocyte-to-monocyte ratio (LMR-PBMC, Fig. 2F) in the samples of the three cohorts. Although the three group of patients were statistically different (Fig. 2E), the cells collected from the early phase COVID-19 group were highly unbalanced towards neutrophils. The NLR-PBMC of 2.1 ± 4.2 (mean ± SD; early phase COVID-19 versus blood donors *t*-test *P* < 0.0001) demonstrated that, in the early phase of the disease, the PBMC fraction was heavily contaminated by neutrophils, while the ratios of the other two groups were much more close to each other. The cells collected from the 14 d post-discharge cohort displayed a mean of 0.13 ± 0.21 (14 d discharged patients versus blood donors *t*-test *P* = 0.0165) whereas the cell ratio from the blood donor group had values ranging from 0.08 ± 0.08. The LMR-PBMC (Fig. 2F) of the control cohort showed a mean of 3.7 ± 1.3, while the mean ratio of cells collected from COVID-19 patients at 24 h were 3.5 ± 2.7 (Welch’s *t*-test vs blood donors *P* = 0.6203) and those from patients 14 days after dismissal were 4.3 ± 2.2 (*t*-test vs healthy controls *P* = 0.0132). Thus indicating that the distribution pattern of mononuclear cells in the density band was similar and the collected fractions contained similar amount of lymphocytes and monocytes. However it is worth to mention that in the present context, the above NLR-PBMC and LMR-PBMC are calculated on recovered cells and provide only a parameter of the cell distribution within the enriched fractions following the separation medium centrifugation.

### Flow cytometry analysis

**Figure 3:**
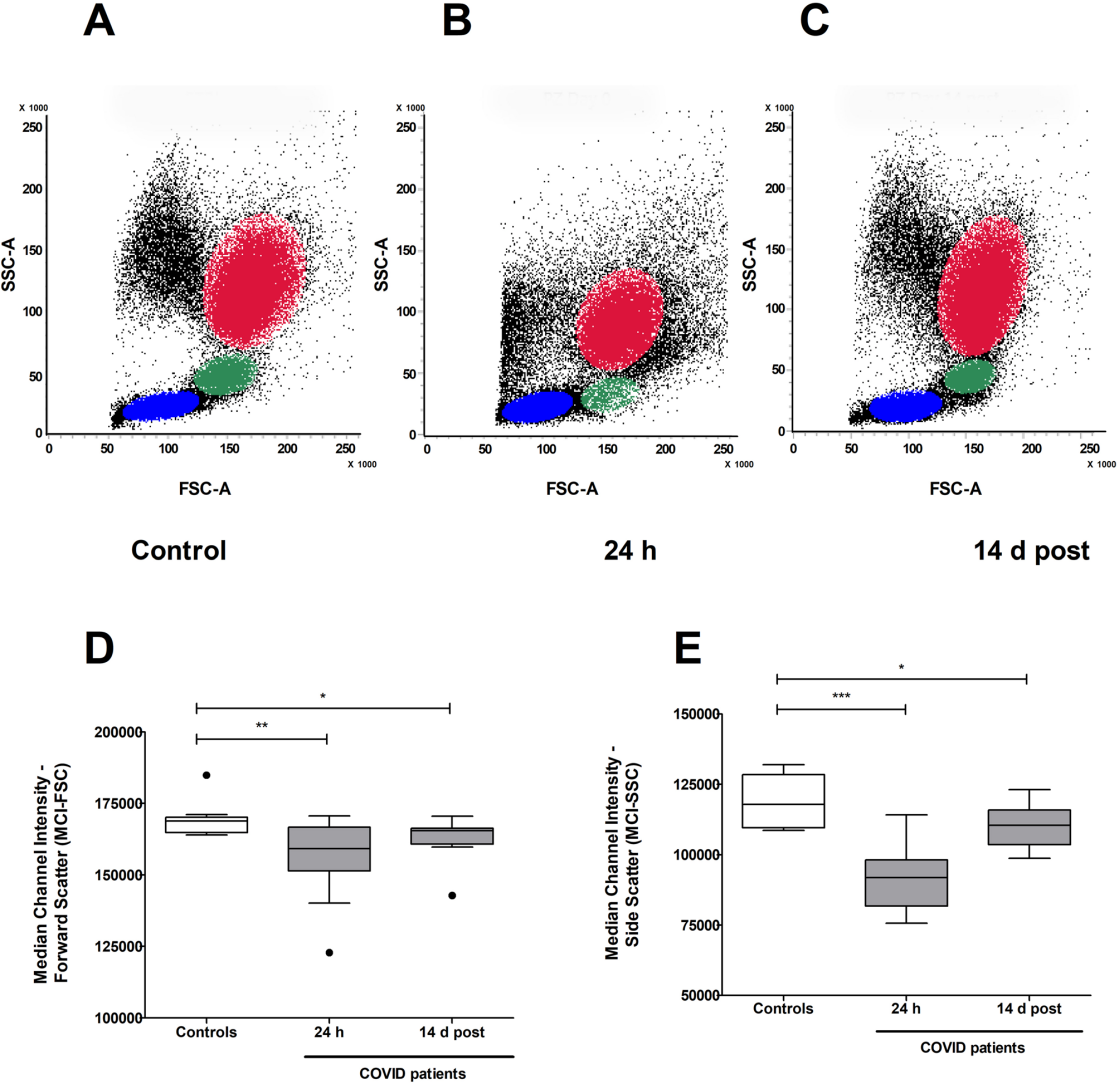
Flow cytometry analysis of peripheral blood leukocyte populations. Granulocytes are shown in red, monocytes in green and lymphocytes in blue. Morphological dot plots are shown, as examples of the different forward scatter (FSC) and side scatter (SSC) properties of the granulocyte population in the different cohort, in panels (**A**–**C**). The median channel intensities of FSC and SSC in the granulocytes gated region of healthy blood donor controls (N = 10), early COVID-19 patients (N = 13) and recovered patients (N = 13) are shown in panels (**D**) and (**E**), respectively. Statistically significant differences were expressed as *for *P* ≤ 0.05, **for *P* ≤ 0.01, ***for *P* ≤ 0.001 and ****for *P* ≤ 0.0001.

Whole blood leftovers were used to analyse the leukocyte population of COVID-19 patients (N = 13) and healthy blood donors (N = 10) by flow cytometry. The cell populations were assessed after red blood cell lysis. Representative forward scatter (FSC) versus side scatter (SSC) dot plots from the three cohorts are depicted in Fig. 3A, B and C. In particular, FSC parameter is proportional to the cells size, while SSC reflects the internal cell complexity (i.e. nuclear morphology and granularity). The leukocyte morphological properties from patient samples taken 24 h from hospitalisation (Fig. 3B) clearly exhibited a shift down in the distribution of the granulocyte population, probably indicating a lack/loss of granularity, when compared to those from both healthy controls (Fig. 3A) and patients at discharge (Fig. 3C). In addition, a diminished number of lymphocytes and an alteration of the monocyte population were observed in the early phase of infection. The post-dismissal specimen (Fig. 3C) showed that the cell distribution pattern returned almost back to normal, though with still a slight increased number of events in the granulocyte region.

The box-and-whisker chart (Fig. 3D) of the median channel intensity (MCI) in the forward scatter (FSC) indicated that there are some significant differences in the cell sizes, indeed the MCI was lower in the 24 h cohort (159,221 MCI, Mann–Whitney U test vs blood donors *P* = 0.0070) and tended to raise again in the 14 d discharged patient group (165,465 MCI, Mann–Whitney U test vs healthy blood donor controls *P* = 0.0236) to levels close to normality (healthy controls median = 168,870 MCI), although not completely back to normal. The most striking difference was observed instead in the granularity (Fig. 3E), where the internal macromolecular complexity of the cells had a massive impact despite the fact that the side scatter (SSC) is measured at a ninety degree angle on the laser light and its signal is weaker than the FSC. The cells from early phase of COVID-19 patients (within 24 h) showed a marked decrease in the SSC compared with the blood donors (U test vs blood healthy controls *P* = 0.0001). The internal complexity was only partially restored 14 d after hospital dismissal (Mann–Whitney test vs blood donors *P* = 0.0378).

### Morphological inspection of COVID-19 samples

**Figure 4:**
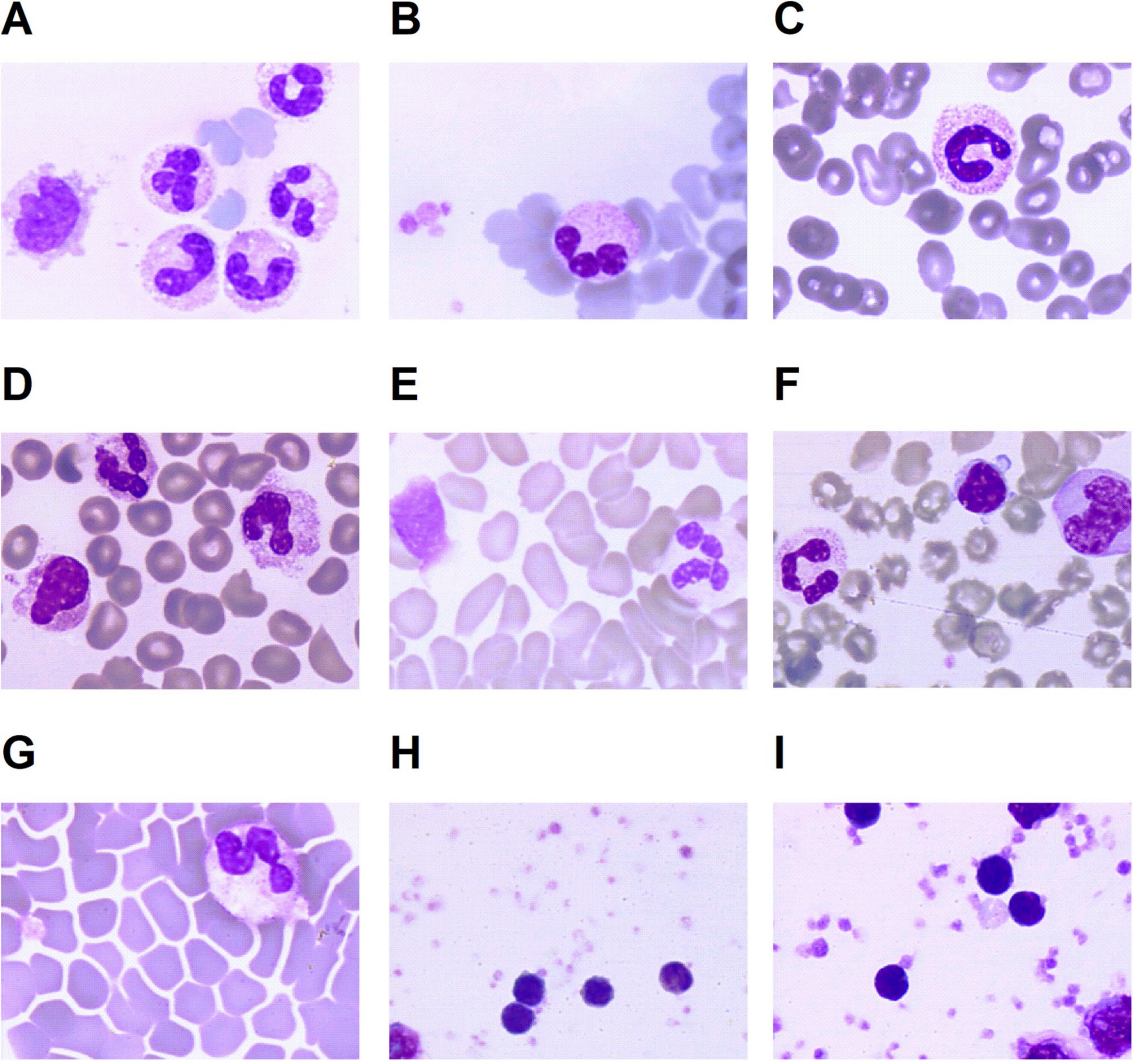
COVID-19 blood smears at different stages and recovered PBMC fractions. Images from blood smears of samples from different patients within 24 h from hospitalisation i.e. early phase COVID-19 are shown in panels (**A**–**D**) while (**E**–**G**) are blood smears from samples taken at day 14 post-discharge of the same patients as (**B**–**D**). Panels (**H**) and (**I**) are examples of samples enriched in mononuclear cells. Magnification for all images was 60×.

Blood smears and PBMCs from density gradients spotted onto glass slides were fixed and stained with May–Grünwald Giemsa for a simple cytology evaluation. In Fig. 4, (panels A–D) we showed the blood smears from four different COVID-19 patients within 24 h from hospitalisation. The samples confirmed the presence of excessive number of mix-shaped neutrophils in the peripheral blood. Some of these neutrophils had either a ribbon-shaped or a horseshoe-shaped nucleus suggesting that these are immature forms. Neutrophils are still present in the blood smears (Fig. 4E, F and G), from specimen taken after 14 days post-discharge, though mostly in the mature form. The panels H and I in Fig. 4 are examples of the mononuclear cell enrichment prior to the last centrifugation to remove cell debris and blood residues.

### Blood cell counts

Almost all the COVID-19 patients and the blood donors had a complete blood count (CBC) test done at the same time or on the same day at which the samples for biobanking were taken. Therefore we have retrieved and retrospectively analysed these data to compare them with the results obtained from the gradient centrifugation.

**Figure 5:**
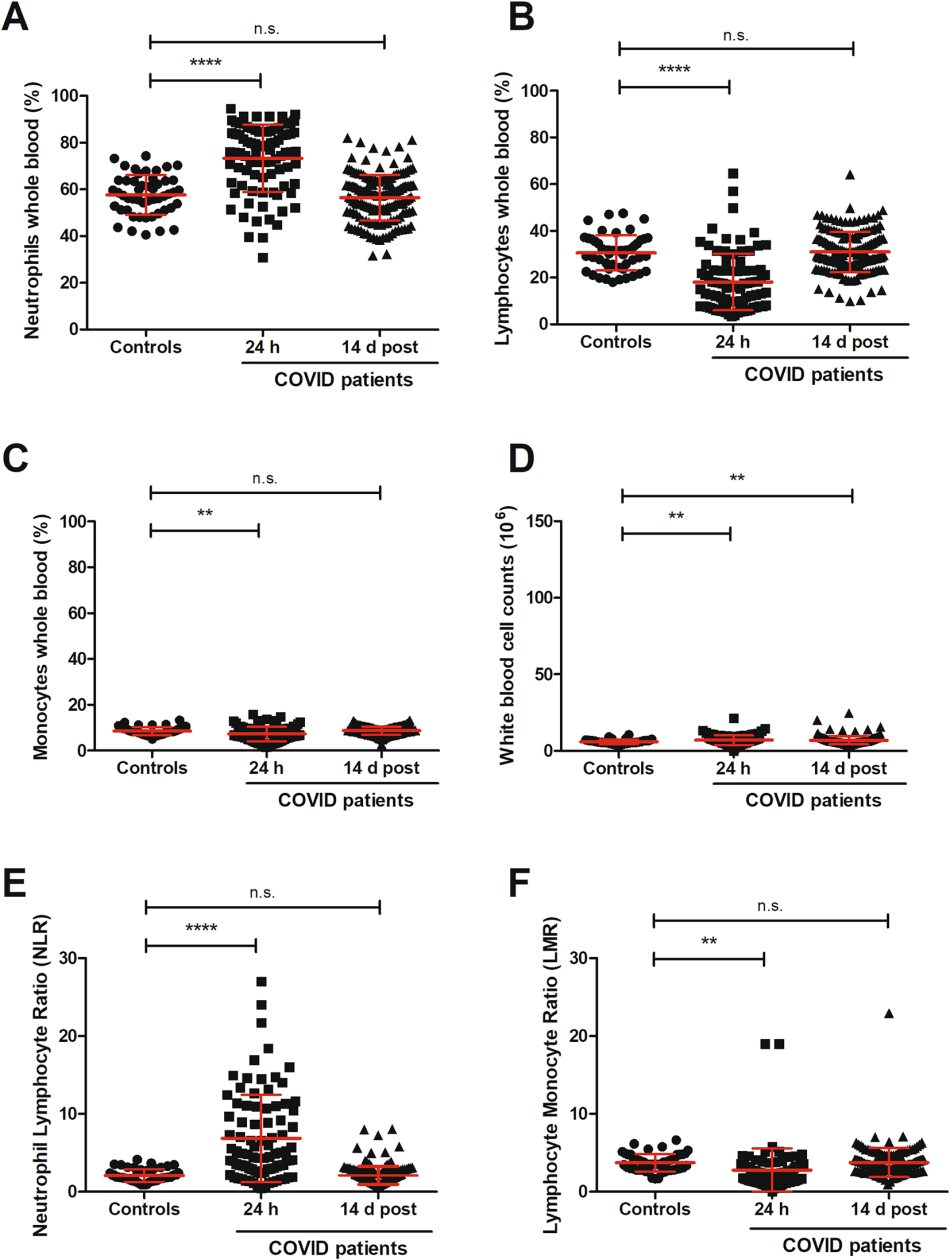
Cell blood count (CBC) test analysis of the different cohorts. CBC tests of healthy controls (N = 50) were performed at the time of blood donations and when research blood was also drawn. Almost all the early stage COVID-19 cohort patients (N = 87) and the recovered patients (N = 170) had also CBC tests carried out at the same time or on the same day when the blood was collected for biobanking. Panel (**A**) represents neutrophil populations of the healthy blood donors, in early stage COVID-19 patients and in the patients of the recovered cohort (14 d post). The lymphocytes from the three cohorts are shown in panel (**B**) while panel (**C**) displays the monocytes. The total cell counts of all three groups are indicated in panel (**D**). The neutrophil-to-lymphocyte ratio (NLR) is displayed in panel (**E**), whereas lymphocyte-to monocyte-ratio (LMR) is shown in panel (**F**). Each dot represents a single patient and the values are expressed as mean ± standard deviation (SD). Statistically significant differences are shown as *for *P* ≤ 0.05, **for *P* ≤ 0.01, ***for *P* ≤ 0.001 and ****for *P* ≤ 0.0001, not statistically significant differences are indicated as n.s.

Early phase COVID-19 patients showed elevated levels of neutrophils i.e. 73.3 ± 14.4% (mean ± SD; Welch’s *t*-test vs blood donors *P* < 0.0001), with a median value of 76.0% (IQR 62.9–84.7) whereas the frequency of neutrophils 14 d post-discharge was 56.4 ± 9.8% (*t*-test vs healthy controls *P* = 0.4141; median 57.6%; IQR 50.4–62.5) and in the whole blood of healthy donors was 57.6 ± 8.5% (median value of 57.7%; IQR 51.2–63.8; Fig. 5A).

The lymphocytes detected at 24 h showed a downward trend (Fig. 5B), confirming the inversion previously seen in the enriched populations (Fig. 2B). These leukocytes displayed a mean of 18.2 ± 12.2% (*t*-test vs blood donors *P* < 0.0001; median 16.1%; IQR 8.1–23.1) early in the SARS-Cov-2 infected individuals which rose to 30.7 ± 7.6% (*t*-test vs healthy controls *P* = 0.8089; median 30.0%; IQR 25.6–36.7) in the discharged patients and was comparable to the values, 31.0 ± 8.6% (median 31.5%; IQR 24.2–36.2), found in the healthy blood volunteers.

The whole blood monocytes showed fewer fluctuations (Fig. 5C) than the enriched fractions in Fig. 3C. The populations were more compact in all three cohorts and displayed mean percentage values of 7.3 ± 3.3 (Welch’s *t*-test vs healthy controls *P* = 0.0055; median 6.8%; IQR 4.9–9.5) at 24 h from admission, with a slight increase in the discharged patients to 8.7 ± 1.9% (*t*-test vs blood donors *P* = 0.5707; median 8.7%; IQR 7.3–10.2) and to 8.5 ± 1.8% (median 8.3%; IQR 7.0–9.6) in the healthy blood donors.

White blood cell (WBC) counts measure the number of leukocytes in the blood and showed minor differences (Fig. 5D). As expected the percentage of the circulating white blood cells was slightly higher in the early phase of the infection 7.1 ± 3.2% (*t*-test vs blood donors *P* = 0.0077; median 6.7%; IQR 4.8–8.8) and in the recovered patients (14 d post-dismissal) 7.0 ± 2.7 (*t*-test vs healthy controls *P* = 0.0010; median 6.4%; IQR 5.5–8.0) while dropped to 6.0 ± 1.5% (median 5.7%; IQR 5.0–6.8) in the healthy blood donor controls.

Since the neutrophil-to-lymphocyte ratio (NLR) and lymphocyte-to-monocyte ratio (LMR) are predictive of inflammatory dysfunctions and have been reported to have prognostic significance of COVID-19 severity, we show here mean ± SD and median with IQR of these biological markers determined in our cohorts (Fig. 5E and F). COVID-19 patients within 24 h from hospitalisation (panel 5E) exhibited NLR levels of 6.9 ± 5.6 (Welch’s *t*-test vs healthy controls *P* < 0.0001; median 4.9; IQR 2.9–10.7), which decreased to 2.2 ± 1.2 (*t*-test vs blood donors *P* = 0.8274; median 1.9; IQR 1.4–2.5) when measured in those samples taken 14 d post-recovery. The latter cohort displayed values similar to the healthy blood donor controls, whose levels were 2.1 ± 0.8 (median 1.8; IQR 1.4–2.7). Conversely LMR levels were higher in the blood donors i.e. 3.7 ± 1.1 (median 3.6; IQR 2.8–4.8) and in the recovered patient samples at 14 days post-discharge 3.8 ± 1.9 (*t*-test vs healthy controls *P* = 0.9123; median 3.5; IQR 2.9–4.2), while in the early COVID-19 samples were 2.8 ± 2.8 (Welch’s *t*-test vs blood donors *P* = 0.0065; median 2.4; IQR 1.4–3.3). Thus both biological marker levels, i.e. NLR and LMR, differed between the groups and were altered in the early phase of the SARS-Cov-2 infection.

## Discussion

Monocytes usually float to a density between 1.079–1.089 g/ml followed by the lymphocytes, therefore the band we collected should have accounted mostly for mono/lymphocyte populations. The cloudy and sticky area might be due to activated monocytes, but also to a massive mobilisation of activated macrophages and mature dendritic cells, which have entered the circulation to reach peripheral tissues. What is the cause that led the erythrocytes to decorate the lymphocytes band is unclear. However, erythrocyte rosetting (E-rosetting) is an immunological reaction occurring between receptors or antibodies on the lymphocyte plasma membrane and epitopes on the erythrocytes, where red blood cells arrange themselves in petal-like flower. Immature B-lymphocytes may be also present in the lymphocyte pool and can express membrane-bound IgD able to capture foreign and self-antigens or to activate other immune effectors. Hemadsorption and hemagglutination are abilities not new to bacteria and parasites (e.g. Treponema pallidum, Plasmodium spp.). Furthermore many viruses, Orthomyxoviridae, Paramyxoviridae, Matonaviridae (i.e. Rubella) among others, can attach to molecules present on the surface of the erythrocytes, thus eventually leading them to agglutinate. CR1/CD35 and CR2/CD21 are complement receptors for C3b/C4b and C3d expressed by several cell populations within the PBMC pool. It is also possible that anti SARS-CoV-2 antibodies, appearing during an early seroconversion, may show some sort of cross-reactivity and recognition of self-epitopes. Indeed, it is worthwhile mentioning that the excessive cell death occurring during the infection may determine the release of unprecedented amount of DNA, histones and cell debris, which in turn could lead to a hyperproliferation of self-reactive lymphocytes and/or, as consequence of extensive cell damage, to the production of autoantibodies.

The abnormal amount of neutrophils collected in the density gradient may be misleading, because we are dealing with isolated PBMCs rather than with the whole blood. However, why neutrophils are present in such numbers in the gradient band remains an open question. They could have been trapped within the complex web of complement components, fibrinogen, fibrin and platelets. Neutrophils might also been kept floating while expelling DNA and proteins, which instead of baiting pathogens, might have been recognised by other immunological effectors e.g. lymphocytes. The excessive formation of NETs may act as parachute, thus keeping most of the neutrophils in suspension, allowing them to float in the density medium rather than sinking and sedimenting on top of the erythrocytes layer. Low density neutrophils are very efficient at generating neutrophil extracellular traps. Intravascular neutrophil aggregation and NET formation may play a pivotal role also in the occlusion of microvessels. The NETs can be responsible for the activation of the vascular endothelium, can promote the vascular damage of endothelial cells, foster platelet aggregation and trigger intravascular coagulation. These low density neutrophils belong to a subpopulation yet not well-defined.

Last, but not least, they might well be exhausted neutrophils after degranulation or, consistent with early stage responses to infection and inflammation where the bone marrow is stimulated to release blood-forming stem cells, immature (low density) neutrophils. The recruitment of immunological effectors at the active site of the infection i.e. the lungs, might have triggered an enhanced mobilisation of immature or naïve neutrophils to compensate the depletion of the circulating primed and activated forms. Incomplete granulopoiesis might confer to immature neutrophils a lower density allowing the flotation onto or within the separation medium.

Density is defined as mass per volume and the blood density is somehow proportional to haematocrit. Changes in the circulating cell populations in COVID-19 patients may affect the total protein concentration of blood, may determine a variation in the blood density and could inhibit an optimal mononuclear cell separation through the density gradient. Therefore the simplest explanation for the modification of the buoyant density of PBMCs is the result of the massive alteration in the cell composition of the circulating blood that occurs in COVID-19 patients.

Although the traits of the outliers were more pronounced and their features appeared, up to a certain extent, amplified, the population dynamics of the cells enriched after fractionation on density medium, traced in essence the distribution pattern of the circulating blood. The ratios, i.e. NLR and LMR, in the whole blood allow a better resolution and provide a better appreciation of the cell distribution. However, despite the fact that the patterns of the ratios above mentioned differed between the groups and appeared to be significant, further studies with a much larger number of samples are required to validate their potential use as predictive markers of the disease severity.

In conclusion, our results show that the infection of SARS-CoV-2 strongly affects the sedimentation of neutrophils on density gradients. The imbalanced recovery of “alleged” mononuclear cells in COVID-19 patients, containing a large amount of LDNs and a diminished number of lymphocytes, confirm what seen by us and others in the whole blood.

## Methods

### Patient population

The study was conducted after obtaining the Fondazione IRCCS Ca’ Granda Ospedale Maggiore Policlinico Ethics Committee approval (COVID-19_Network IRB #241_2020) and the patient informed consents for research studies and biobanking. All methods were performed in accordance with the relevant guidelines and regulations.

Two hundred eighty-one samples were collected and harvested at the Processing Facility and Biobank POLI-MI from April the 1st until April the 30th 2020, during the SARS-CoV-2 pandemic. Seventeen samples gave insufficient material or the patients had duplicate samples, and have been excluded from the data analysis. In that period we received samples from two hundred seventy patients ranging between 24 and 99 years of age and, as some progressed towards the recovery, for ten patients we had also follow-up samples. All the patients were diagnosed with COVID-19, had SARS-CoV-2 positive nasopharyngeal swab detected through real-time reverse transcription–polymerase chain reaction (real-time RT-PCR) and were hospitalised in the different COVID-19 Units of IRCCS Ca’ Granda Ospedale Maggiore Policlinico. Blood samples were taken either within 24 h from the admission into the Unit and/or at day 14 after hospital discharge.

The healthy control samples were kindly provided by registered blood donors visiting our Blood Transfusion Centre for their regular blood donations (after written informed consent and within the approved protocol CoDS).

Demographics of the patients and of the blood donors are shown in Table 1.

**Table 1:** Patient and blood donor demographics.

Parameter	COVID-19 +			Blood donors
	24 h	14 d	Total	
Number of patients	101	179	270*	50
Number of samples	102	179	281	50
Male (%)	55	67.8	64.4	68
Age (median)	64	54	57	45.8

A total number of 90 early phase COVID-19 and 174 COVID-19 recovered patients were finally included in the analysis. *Ten of patients had follow-up samples i.e. at the admission and 14 days post-discharge.

### Isolation of peripheral blood mononuclear cells

Peripheral blood samples were drawn into EDTA vacutainer tubes. PBMCs were isolated by polysaccharide density gradient centrifugation (Lymphoprep™—Axis-Shield Alere Technologies AS, Oslo, Norway) as described previously^63^ with minor modifications. Briefly, after centrifugation at 1560 g for 10 min at 20 °C, the plasma was collected and snap-frozen in LN_2_ for further studies. The remaining buffy coats (i.e. leukocyte concentrates) from the same patient samples were pooled together and then diluted 1:2 (vol:vol) in 0.9% sodium chloride injection solution, USP (Baxter S.p.A., Rome, Italy) prior to be layered onto the density gradient medium. The samples were centrifuged at 900 g for 20 min without brake. PBMC isolation procedure was performed at 20 °C to avoid any possible variation in the density of the density gradient medium. The PBMC rings were collected, resuspended and washed twice with saline by centrifugation at 450 g and 350 g, respectively. Cell pellets were suspended again in freezing medium for storage.

### Cell populations and flow cytometry analysis

Cell counts were performed by running the samples through an automated haematology cell analyser (Sysmex XN-1000™ DASIT S.p.A., Cornaredo, Italy), in body fluid or complete blood count (CBC; for the clinical samples) mode.

Peripheral blood samples leftover were analysed within 24 h from the withdrawal, after lysing the erythrocytes with lysing solution (BD Pharm Lyse™, BD Biosciences). After washing, the samples were acquired with a BD FACSLyric flow cytometer equipped a 405 nm violet laser, a 488 nm blue laser and a 647 nm red laser. For each tube 50,000 events in the lymphocyte gate, forward scatter (FSC) versus side scatter (SSC), were acquired and the data were analysed using FACSSuite software (BD Biosciences). An automatic standard setup was applied for each acquisition. Internal quality assurance procedures included BD cytometer setup and tracking beads, according to the manufacturer’s instructions.

### May–Grünwald Giemsa staining

Whole blood smears and cell suspensions spotted onto glass slides were air dried under the biological safety cabinet class II and fixed in 100% methanol for 30 min prior to staining. The slides were stained with May-Grünwald working solution for 15 min, rinsed with water, immerged in Giemsa working solution for 20 min, rinse thoroughly with water and air dried. The glass slides were imaged with an Olympus BX S3 microscope equipped with a Sony CCD 3 camera, objective magnification at 10×, 20×, 40×, 60× oil and 100× oil. All images were acquired with Sysmex LAFIA, version 2, blood image filing software.

### Statistical analysis

All graphs display mean and standard deviation (SD). Statistical analysis was performed using the Welch’s *t*-test, for unequal sample sizes, or Mann–Whitney U test and one-way ANOVA to calculate statistically significant differences and individual P values using GraphPad Prism version 5.00 (GraphPad Software, San Diego California, USA, www.graphpad.com). Statistically significant differences were expressed as *for *P* ≤ 0.05, **for *P* ≤ 0.01, ***for *P* ≤ 0.001 and ****for *P* ≤ 0.0001, not statistically significant differences are indicated as n.s. for *P* > 0.05.

## Data Availability

The datasets generated during the current study are available from the corresponding author on reasonable request.
